# Epidemiology, risk factors, and clinical impact of early post-transplant infection in older kidney transplant recipients: the Korean organ transplantation registry study

**DOI:** 10.1186/s12877-020-01859-3

**Published:** 2020-12-02

**Authors:** Jin Sug Kim, Kyung Hwan Jeong, Dong Won Lee, Sam Yeol Lee, Sang Ho Lee, Jaeseok Yang, Curie Ahn, Hyeon Seok Hwang, the Korean Organ Transplantation Registry Study Group, Curie Ahn, Jaeseok Yang, Jin Min Kong, Oh. Jung Kwon, Deok Gie Kim, Cheol-Woong Jung, Yeong Hoon Kim, Joong Kyung Kim, Chan-Duck Kim, Ji Won Min, Sung Kwang Park, Yeon Ho Park, Park Jae Berm, Jung Hwan Park, Jong-Won Park, Tae Hyun Ban, Sang Heon Song, Seung Hwan Song, Ho Sik Shin, Chul Woo Yang, Hye Eun Yoon, Kang Wook Lee, Dong Ryeol Lee, Dong Won Lee, Sam Yeol Lee, Sang-Ho Lee, Su Hyung Lee, Jung Jun Lee, Lee Jung Pyo, Jeong-Hoon Lee, Jin Seok Jeon, Heungman Jun, Kyung Hwan Jeong, Ku Yong Chung, Hong Rae Cho, Ju Man Ki, Dong-Wan Chae, Soo Jin Na Choi, Duck Jong Han, Seungyeup Han, Kyu Ha Huh

**Affiliations:** 1 Division of Nephrology, Department of Internal Medicine, College of Medicine, Kyung Hee University, Seoul, Republic of Korea; 2 Division of Nephrology, Department of Internal Medicine, Pusan National University School of Medicine, Busan, Republic of Korea; 3 Department of Surgery, Kangdong Sacred Heart Hospital, Hallym University College of Medicine, Seoul, Republic of Korea; 4 Department of Surgery, Seoul National University Hospital, Seoul, Republic of Korea; 5 Department of Nephrology, Seoul National University Hospital, Seoul, Republic of Korea

**Keywords:** Kidney transplantation, Older kidney recipient, Infectious complication

## Abstract

**Background:**

As in younger recipients, post-transplant infection is a frequent and devastating complication after kidney transplantation (KT) in older recipients. However, few studies have analyzed characteristics of post-transplant infection in older kidney recipients. In this study of a nation-wide cohort of older kidney recipients, we investigated the current epidemiology, risk factors, and clinical impacts of early post-transplant infection, which was defined as infectious complications requiring hospitalization within the first 6 months after KT.

**Methods:**

Three thousand seven hundred thirty-eight kidney recipients registered in the Korean Organ Transplantation Registry between 2014 and 2017 were enrolled. Recipients were divided into two groups, younger (*n* = 3081) and older (*n* = 657), with a cutoff age of 60 years. We observed characteristics of early post-transplant infection, and investigated risk factors for the development of infection. We also analyzed the association of early post-transplant infection with clinical outcomes including cardiac events, rejection, graft loss, and all-cause mortality.

**Results:**

The incidence of early post-transplant infection was more frequent in older recipients (16.9% in younger group and 22.7% in older group). Bacteria were the most common causative pathogens of early post-transplant infection, and the most frequent site of infection was the urinary tract in both older and younger recipients. Older recipients experienced more mycobacterial infections, co-infections, and multiple site infections compared with younger recipients. In older recipients, female sex (HR 1.398, 95% CI 1.199–1.631), older donor age (HR 1.010, 95% CI 1.004–1.016), longer hospitalization after KT (HR 1.010, 95% CI 1.006–1.014), and experience of acute rejection (HR 2.907, 95% CI 2.471–3.419) were independent risk factors for the development of early post-transplant infection. Experiencing infection significantly increases the incidence of rejection, graft loss, and all-cause mortality.

**Conclusion:**

Our results illustrate current trends, risk factors, and clinical impacts of early post-transplant infection after KT in older recipients. Considering the poor outcomes associated with early post-transplant infection, careful screening of recipients at high risk for infection and monitoring of recipients who experience infection are advised. In addition, since older recipients exhibit different clinical characteristics than younger recipients, further studies are needed to establish effective strategies for treating older recipients.

**Supplementary Information:**

**Supplementary information** accompanies this paper at 10.1186/s12877-020-01859-3.

## Backgroud

Kidney transplantation (KT) is a preferred renal replacement therapy for patients with end-stage renal disease (ESRD) [1]. Although short- and long-term clinical outcomes of KT have greatly improved, post-transplant infection remains a devastating complication after KT [2]. Post-transplant infection is one of major causes of mortality and graft loss among kidney recipients [3]. It has been reported that 40–80% of kidney recipients experience infectious complications after KT [4, 5]. Post-transplant infections exhibit various patterns over time after KT. The characteristics of post-transplant infection have changed due to diverse factors such as new surgical techniques, use of prophylactic antibiotics, and changes in immunosuppressant type [6, 7]. Life-threatening infections are more likely to occur within the first 6 months after transplantation, which is assumed to be associated with the use of high-dose immunosuppressants during this time [4, 6]. Therefore, studies on infectious complications occurring during the early post-transplant period are needed.

In recent years, the number of older patients with ESRD has increased, and the proportion of the elderly among kidney recipients has also increased [8, 9]. Older kidney recipients are at high risk for post-transplant infection due to conditions including immunosuppressant use, multiple comorbidities, frailty, and immune senescence [9–12]. However, previous studies of KT in the elderly have focused on traditional outcomes such as rejection and survival, and few studies have evaluated infectious complications after KT in the elderly [12, 13].

In this study, we investigated current incidence, causative pathogens, and sites of overall early post-transplant infection, which was defined as infectious complications requiring hospitalization within the first 6 months after KT in older kidney recipients using a well-organized nationwide cohort. We additionally analyzed risk factors for the development of early post-transplant infection and its impacts on clinical outcomes.

## Methods

### Study population and design

The study sample was derived from the Korean Organ Transplantation Registry (KOTRY) database established by the Korean Society for Transplantation. The KOTRY is a prospective, multicenter, nationwide cohort study that includes data for five types of solid organ transplantation in Korea: kidney, liver, heart, lung, and pancreas. Further details of the KOTRY have been previously described [14].

We analyzed data for 3738 kidney recipients registered in the KOTRY database between April 2014 and December 2017. The recipients were divided into two groups according to age (younger recipients < 60 years-old, *n* = 3081; older recipients ≥60 years-old, *n* = 657). We observed the incidence, causative pathogens, and sites of early post-transplant infection, defined as infectious complications requiring hospitalization within the first 6 months after KT. Infectious complications that occurred during the hospitalization period after kidney transplantation were excluded. If several different infections occurred in one patient, they were counted as separate cases. We also analyzed risk factors for early post-transplant infection and impacts on clinical outcomes by comparing recipients who experienced infections with those who did not.

All study procedures complied with the principles expressed in the Declaration of Helsinki. The study protocol was reviewed and approved by the Institutional Review Board of each center, and the approval number from Kyung Hee University Medical Center is 2019–11-074.

### Variables, study outcomes, and definitions

Data for variables including age, sex, cause of ESRD, duration of under dialysis before KT, underlying diseases, and laboratory findings were collected for every recipient. Transplant-related data, such as age and sex of donor, whether the donor was deceased or not, ABO incompatibility, presence of donor specific antibody (DSA), and type of immunosuppressive drugs, were also recorded.

As described previously, early post-transplant infection was defined as infectious complications requiring hospitalization within the first 6 months of discharge after receiving KT. Infectious episodes were categorized according to microbiologic etiologies or sites of infection. The microbiologic etiologies included bacteria, virus, mycobacteria, fungus, and pneumocystis pneumonia (PCP). Infectious episodes without identified causative pathogens were classified as not identified, and cases that were concurrently infected with two or more types of pathogens were classified as co-infections. The sites of infection were determined as wound, allograft, central nervous system (CNS) and eye, respiratory tract, gastrointestinal (GI) system and liver, urinary tract, vascular system, skin and musculoskeletal system, blood stream, others, multiple sites, and unknown. Cases that were infected at two or more sites were classified as multiple sites, and others include viral antigenemia and DNAemia.

The primary outcomes of this study were clinical characteristics of early post-transplant infection, including incidence, causative pathogens, and site of infection in older kidney recipients. The secondary outcomes were risk factors of early post-transplant infection and clinical outcomes after infection in terms of cardiac events, rejection, graft loss, and all-cause mortality. Cardiac events included myocardial infarction, ischemic heart disease, new-onset congestive heart failure, arrhythmia, and death due to cardiovascular disease. Rejection included both clinical rejection and biopsy-proven rejection. Graft loss was defined as dependence on dialysis lasting more than 3 months. All-cause mortality included both cardiac death and non-cardiac death.

### Statistics

Continuous variables were analyzed by Student’s t-test and presented as mean ± standard deviation (SD). Analyses of categorical data were performed using chi-square or Fisher’s exact tests and reported as frequencies and percentages. Multiple Cox regression analyses determine the independent association of variables with early post-transplant infection, after adjusting for several confounders. The results are presented as hazard ratios (HR) ± 95% confidence intervals (CI), and statistical significance is indicated. Survival curves were estimated by the Kaplan–Meier method and compared with the log-rank test. All statistical analyses were performed using SPSS software version 19.0 (SPSS Inc., Chicago, IL, USA). A *p* value < 0.05 was considered to be statistically significant.

## Results

### Clinical characteristics of the study sample

Clinical characteristics, laboratory findings, and transplant-related data are summarized in Table 1. Among the 3738 kidney recipients included in this study, 657 (17.6%) were older than 60 years old age. There were significant differences in sex, body mass index (BMI), and cause of ESRD between the two groups. The prevalences of diabetes mellitus, hypertension, and cardiovascular disease were significantly greater in the older group. Transplant-related data, age of donor, whether donor was deceased or not, desensitization, length of hospitalization after KT, and type of induction immunosuppression were significantly different between groups. The mean follow-up duration after KT was 27. Ninety-five months in the younger group and 25.14 months in the older group (*p* <  0.001). Overall, 670 (17.9%) recipients experienced infectious complications requiring hospitalization within the first 6 months after KT. The incidence of early post-transplant infection was significantly more frequent in the older group (16.9% in younger group and 22.7% in older group, respectively, *p* <  0.001).

**Table 1:** Baseline characteristics of study sample

	Overall (*N* = 3738)	< 60 year-old (*n* = 3081)	≥ 60 year-old (*n* = 657)	p
Age (years)	48.78 ± 11.50	45.53 ± 9.88	64.08 ± 3.48	< 0.001
Sex, female (%)	1527 (40.9%)	1287 (41.8%)	240 (36.5%)	0.013
Body mass index (kg/m^2^)	23.05 ± 3.52	22.97 ± 3.61	23.41 ± 3.05	0.001
Cause of ESRD, n (%)				< 0.001
Diabetes mellitus	870 (23.3%)	642 (20.8%)	228 (34.7%)	
Hypertension	612 (16.4%)	486 (15.8%)	126 (19.2%)	
Glomerulonephritis	1251 (33.5%)	1121 (36.4%)	130 (19.8%)	
ADPKD	172 (4.6%)	137 (4.4%)	35 (5.3%)	
Others	123 (3.3%)	103 (3.3%)	20 (3.0%)	
Unknown	710 (19.0%)	592 (19.2%)	118 (18.0%)	
Duration of dialysis (months)	56.17 ± 64.13	47.29 ± 62.77	50.73 ± 61.03	0.201
Diabetes mellitus, n (%)	1073 (28.7%)	778 (25.3%)	295 (44.9%)	< 0.001
Hypertension, n (%)	3334 (89.2%)	2719 (88.3%)	615 (93.6%)	< 0.001
Cardiovascular disease, n (%)	404 (10.8%)	260 (8.4%)	144 (22.0%)	< 0.001
Hemoglobin (g/dL)	10.73 ± 1.58	10.70 ± 1.59	10.88 ± 1.52	0.006
Creatinine, baseline (mg/dL)	8.79 ± 3.36	8.96 ± 3.42	8.01 ± 2.98	< 0.001
Creatinine, discharge (mg/dL)	1.22 ± 0.78	1.22 ± 0.79	1.21 ± 0.69	0.708
Albumin (g/dL)	3.93 ± 0.52	3.93 ± 0.53	3.89 ± 0.49	0.049
Re-transplantation, n (%)	289 (7.7%)	249 (8.1%)	40 (6.1%)	0.082
Donor age (years)	46.44 ± 13.18	45.47 ± 12.78	50.95 ± 14.05	< 0.001
Donor sex, female (%)	1720 (46.0%)	1437 (46.7%)	283 (43.1%)	0.095
Deceased donor, n (%)	1446 (38.7%)	1111 (36.1%)	335 (51.0%)	< 0.001
Desensitization, n (%)	845 (22.6%)	723 (23.5%)	122 (18.6%)	0.006
ABO incompatibility, n (%)	586 (15.7%)	495 (16.1%)	91 (13.9%)	0.156
Presence of DSA, n (%)	230 (6.2%)	184 (6.0%)	46 (7.0%)	0.319
Length of hospitalization after KT (days)	17.88 ± 10.63	17.53 ± 10.35	19.56 ± 11.71	< 0.001
Induction immunosuppression				< 0.001
No use, n (%)	66 (1.8%)	52 (1.7%)	14 (2.1%)	
Basiliximab, n (%)	2924 (78.2%)	2447 (79.4%)	477 (72.8%)	
ATG, n (%)	668 (17.9%)	513 (16.7%)	155 (23.7%)	
Basiliximab + ATG, n (%)	77 (2.1%)	68 (2.2%)	9 (1.4%)	
Calcineurin inhibitors, n (%)				0.067
No use	45 (1.2%)	32 (1.0%)	13 (2.0%)	
Tacrolimus	3568 (95.5%)	2952 (95.8%)	616 (94.0%)	
Cyclosporine	122 (3.3%)	96 (3.1%)	26 (4.0%)	
Mycophenolate, n (%)	3399 (90.9%)	2819 (91.5%)	580 (88.7%)	0.021
mTOR inhibitors, n (%)	48 (1.3%)	37 (1.2%)	11 (1.7%)	0.324
Steroid, n (%)	3647 (97.6%)	3011 (97.2%)	636 (97.2%)	0.430
Follow-up duration (months)	27.45 ± 12.47	27.95 ± 12.41	25.14 ± 12.49	< 0.001
Early post-transplant infection, n (%)	670 (17.9%)	521 (16.9%)	149 (22.7%)	< 0.001

*ESRD* end stage renal disease, *ADPKD* autosomal dominant polycystic kidney disease, *DSA* donor specific antibody, *KT* kidney transplantation, *ATG* anti-thymocyte globulin

### Characteristics of early post-transplant infection in kidney recipients

**Fig. 1:**
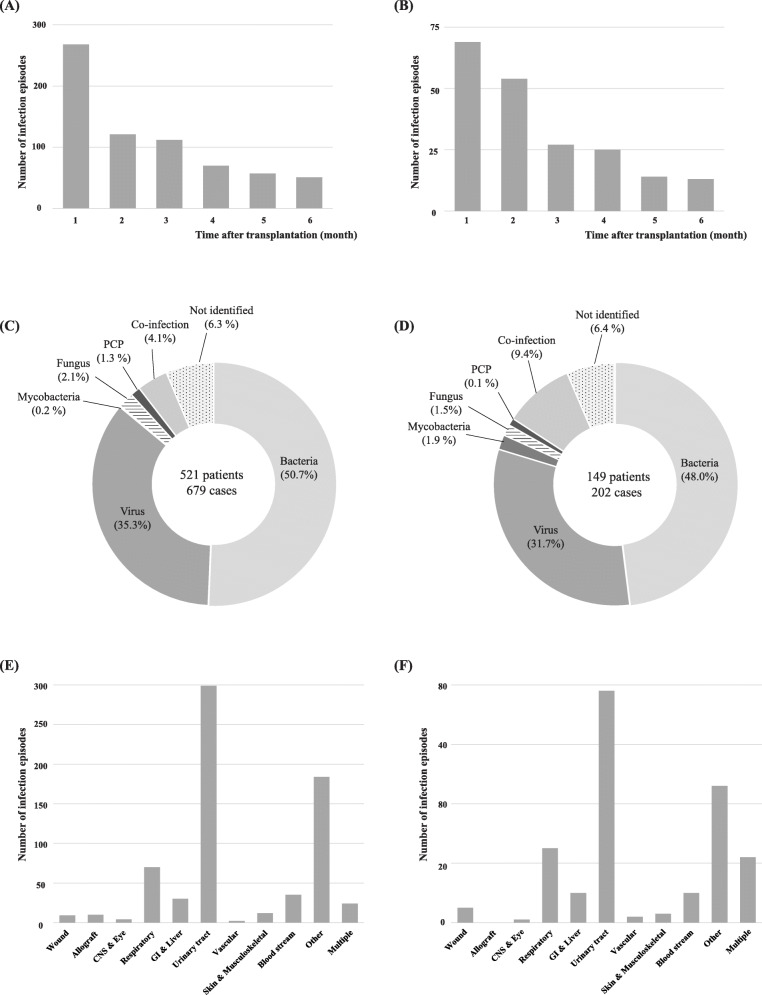
Frequencies of early post-transplant infection after KT according to time (**a**, **b**), causative pathogens (**c**, **d**), and sites (**e**, **f**). **a**, **c**, and **e** represent younger recipients and **b**, **d**, and **f** represent older recipients. KT, kidney transplantation; PCP, pneumocystis pneumonia; CNS, central nervous system; and GI system, gastrointestinal system

Figure 1 shows frequencies, causative pathogens, and sites of early post-transplant infection. As shown in Fig. 1a and b, episodes of infection were more frequent in the first month after KT, and the frequency tended to decrease with time in both groups. During the first 6 months after KT, the most common causative pathogen was bacteria, followed by viruses in both groups. Compared with the younger group, the older group experienced more mycobacterial infections and co-infections (Fig. 1c and d). The distributions of infection episodes according to infectious sites are shown in Fig. 1e and f. The urinary tract was the most frequent site of early post-transplant infection in both groups. Older recipients experienced more multiple site infections compared with younger recipients.

### Risk factors for early post-transplant infection

We conducted univariate and multivariate Cox regression analyses to identify risk factors associated with development of early post-transplant infection. Variables with *p*-values < 0.10 in the univariate analysis were included for multivariate analysis. In the younger group, female sex of the recipient (HR 1.323, 95% CI, 1.103–1.587, *p* = 0.003), history of CVD (HR 1.454, 95% CI, 1.108–1.906, *p* = 0.007), deceased donor (HR 1.409, 95% CI, 1.120–1.772, p = 0.003) older donor age (HR 1.008, 95% CI, 1.001–1.015, *p* = 0.002), presence of DSA (HR 1.427, 95% CI 1.016–2.006, *p* = 0.040), longer hospitalization after KT (HR 1.015, 95% CI, 1.006–1.017, *p* <  0.001), and experience of acute rejection before infection (HR 2.637, 95% CI, 2.455–3.426, p <  0.001) were independent risk factors for the development of early post-transplant infection (Table 2). In the older group, female sex of the recipient (HR 1.398, 95% CI, 1.199–1.631, *p* <  0.001), deceased donor (HR 1.364, 95% CI, 1.137–1.637, *p* = 0.001), older donor age (HR 1.010, 95% CI, 1.004–1.016, p = 0.001), longer hospitalization after KT (HR 1.010, 95% CI, 1.006–1.014, *p* = 0.016), and experience of acute rejection before infection (HR 2.907, 95% CI,2.471–3.419, p <  0.001) were independent risk factors for the development of early post-transplant infection (Table 3).

**Table 2:** Risk factors for the development of early post-transplant infection in younger recipients

< 60 year-old	Univariate		Multivariate	
	HR (95% CI)	p	HR (95% CI)	p
Sex, female	1.276 (1.074–1.515)	0.006	1.323 (1.103–1.587)	0.003
BMI (kg/m^2^)	1.017 (0.993–1.041)	0.173		
Cause of ESRD				
Diabetes mellitus	1			
Hypertension	1.007 (0.756–1.341)	0.964		
Glomerulonephritis	1.007 (0.796–1.275)	0.952		
ADPKD	1.100 (0.712–1.700)	0.666		
Others	1.209 (0.761–1.921)	0.421		
Unknown	0.913 (0.691–1.205)	0.518		
Duration of dialysis (month)	1.002 (1.001–1.003)	< 0.001	1.000 (0.999–1.002)	0.377
Diabetes mellitus	1.015 (0.834–1.237)	0.879		
Hypertension	1.021 (0.779–1.336)	0.882		
Cardiovascular disease	1.520 (1.167–1.980)	0.002	1.454 (1.108–1.906)	0.007
Creatinine, baseline (mg/dL)	1.004 (0.979–1.030)	0.761		
Creatinine, discharge (mg/dL)	1.015 (0.916–1.125)	0.772		
Re-transplantation	1.065 (0.782–1.450)	0.690		
Deceased donor	1.553 (1.306–1.846)	< 0.001	1.409 (1.120–1.772)	0.003
Donor age (year)	1.010 (1.003–1.017)	0.004	1.008 (1.001–1.015)	0.022
Donor sex, female	1.346 (1.129–1.604)	0.001	1.192 (0.992–1.433)	0.061
Desensitization	1.015 (0.829–1.243)	0.883		
ABO incompatibility	0.757 (0.586–0.979)	0.034	0.934 (0.708–1.230)	0.626
Presence of DSA	1.536 (1.128–2.092)	0.006	1.427 (1.016–2.006)	0.040
Length of hospitalization after KT (day)	1.015 (1.011–1.020)	< 0.001	1.011 (1.006–1.017)	< 0.001
Induction immunosuppression				
No use	1		1	
Basiliximab	2.226 (0.831–5.959)	0.111	2.511 (0.479–8.252)	0.051
ATG	2.760 (1.016–7.493)	0.046	2.589 (0.952–7.042)	0.062
Basiliximab + ATG	2.894 (0.953–8.793)	0.061	2.337 (0.767–7.118)	0.135
Calcineurin inhibitors				
No use	1			
Tacrolimus	1.345 (0.503–3.597)	0.555		
Cyclosporine	1.146 (0.377–3.481)	0.810		
Steroid	1.477 (0.735–2.970)	0.273		
Acute rejection	2.993 (2.499–3.585)	< 0.001	2.637 (2.455–3.426)	< 0.001

*BMI* body mass index, *ESRD* end stage renal disease, *ADPKD* autosomal dominant polycystic kidney disease, *DSA* donor specific antibody, *KT* kidney transplantation, *ATG* anti-thymocyte globulin

**Table 3:** Risk factors for the development of early post-transplant infection in older recipients

≥ 60 year-old	Univariate		Multivariate	
	HR (95% CI)	p	HR (95% CI)	p
Sex, female	1.548 (1.119–2.142)	0.008	1.398 (1.199–1.631)	< 0.001
BMI (kg/m^2^)	0.991 (0.939–1.046)	0.741		
Cause of ESRD				
Diabetes mellitus	1			
Hypertension	0.898 (0.554–1.454)	0.661		
Glomerulonephritis	1.192 (0.767–1.854)	0.436		
ADPKD	0.781 (0.334–1.823)	0.568		
Others	1.135 (0.452–2.850)	0.787		
Unknown	1.226 (0.778–1.932)	0.379		
Duration of dialysis (month)	1.003 (1.001–1.005)	0.004	1.001 (1.000–1.002)	0.156
Diabetes mellitus	0.880 (0.635–1.219)	0.441		
Hypertension	0.663 (0.375–1.172)	0.157		
Cardiovascular disease	0.931 (0.627–1.384)	0.725		
Creatinine, baseline (mg/dL)	0.988 (0.935–1.045)	0.680		
Creatinine, discharge (mg/dL)	1.153 (0.945–1.408)	0.161		
Re-transplantation	0.994 (0.520–1.901)	0.986		
Deceased donor	1.495 (1.076–2.077)	0.017	1.364 (1.137–1.637)	0.001
Donor age (year)	1.022 (1.009–1.034)	0.001	1.010 (1.004–1.016)	0.001
Donor sex, female	1.204 (0.864–1.677)	0.273		
Desensitization	1.257 (0.849–1.859)	0.253		
ABO incompatibility	1.050 (0.662–1.667)	0.836		
Presence of DSA	1.324 (0.749–2.340)	0.334		
Length of hospitalization after KT (day)	1.016 (1.008–1.025)	< 0.001	1.010 (1.006–1.014)	< 0.001
Induction immunosuppression				
No use	1		1	
Basiliximab	1.513 (0.373–6.134)	0.562	2.051 (0.916–4.593)	0.081
ATG	2.022 (0.489–8.357)	0.331	2.131 (0.941–4.827)	0.070
Basiliximab + ATG	5.087 (0.987–26.223)	0.052	2.138 (0.851–5.370)	0.106
Calcineurin inhibitors				
No use	1			
Tacrolimus	3.285 (0.460–23.478)	0.236		
Cyclosporine	0.495 (0.031–7.913)	0.619		
Steroid	0.974 (0.361–2.631)	0.958		
Acute rejection	3.377 (2.399–4.754)		2.907 (2.471–3.419)	< 0.001

*BMI* body mass index, *ESRD* end stage renal disease, *ADPKD* autosomal dominant polycystic kidney disease, *DSA* donor specific antibody, *KT* kidney transplantation, *ATG* anti-thymocyte globulin

### Clinical outcomes according to the presence of early post-transplant infection

**Fig. 2:**
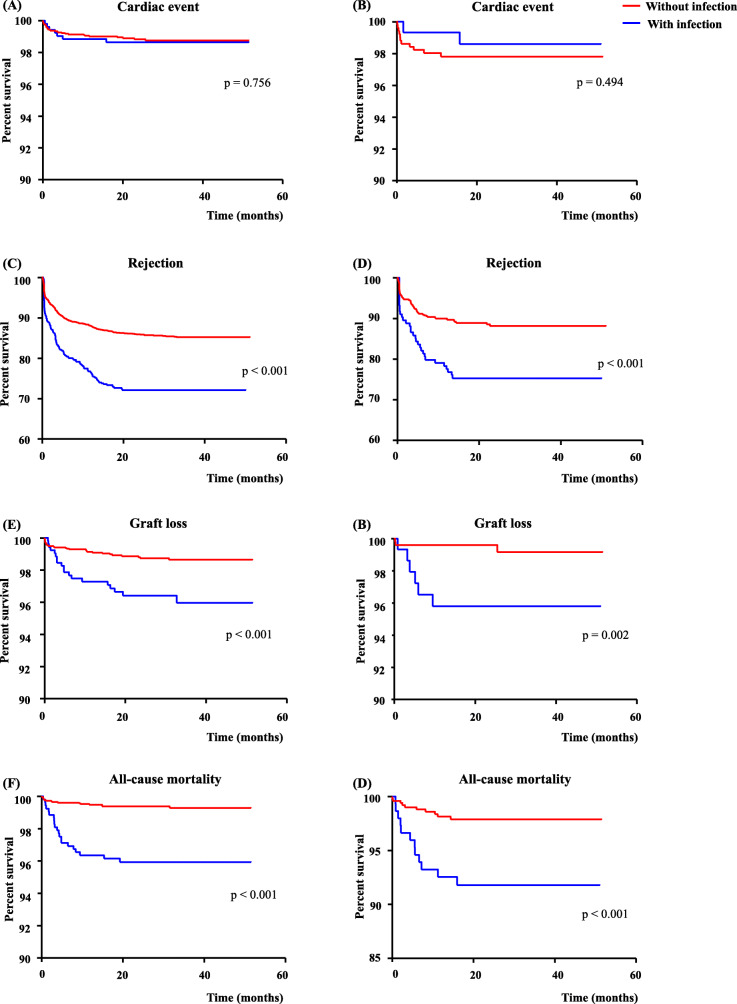
Event free survival according to early post-transplant infection after kidney transplantation. **a**, **c**, **e**, and **g** represent younger recipients and **b**, **d**, **f**, and **h** represent older recipients

We analyzed clinical outcomes after post-transplant infection including cardiac events, rejection, graft loss, and all-cause mortality. Table 4 shows the clinical outcomes of the two groups according to the presence of early post-transplant infection. Overall, 49 (1.3%), 578 (15.5%), and 58 (1.6%) recipients experienced episodes of cardiac events, rejection, and graft loss, respectively, during the follow-up period. The number of cases of all-cause mortality during the follow-up period was 59 (1.6%). These clinical outcomes were more likely to occur in recipients with early post-transplant infection. Figure 2 shows event-free survival according to the presence of early post-transplant infection in both groups. In both group, recipients who experienced early post-transplant infection had lower event-free survival rates than those who did not in cases of rejection (72.1% vs. 85.2%, *p* <  0.001; 75.1% vs. 88.1%, p <  0.001, respectively), graft loss (95.9% vs. 98.6%, p <  0.001; 95.8% vs. 99.2%, *p* = 0.002, respectively), and all-cause mortality (95.9% vs. 99.3%, p <  0.001; 91.8% vs. 97.9%, p <  0.001, respectively). However, early post-transplant infection did not significantly affect the occurrence of cardiac events in either group (98.6% vs. 98.7%, *p* = 0.756; 98.6% vs. 97.8%, *p* = 0.494, respectively). These associations between early post-transplant infection and clinical outcomes maintained a similar pattern even after adjusting for covariates in the analyses (Supplementary Table 1).

**Table 4:** Clinical outcomes according to the presence or absence of early post-transplant infection

	Overall (N = 3738)	< 60 year-old (n = 3081)		≥ 60 year-old (n = 657)	
		With infection (***n*** = 521)	Without infection (***n*** = 2560)	With infection (***n*** = 149)	Without infection (***n*** = 508)
**Cardiac events (n, %)**	49 (1.3%)	7 (1.3%)	29 (1.1%)	2 (1.3%)	11 (2.2%)
**Rejection (n, %)**	578 (15.5%)	133 (25.5%)	354 (13.8%)	34 (22.8%)	57 (11.2%)
**Graft loss (n, %)**	58 (1.6%)	19 (3.6%)	30 (1.2%)	6 (4.0%)	3 (0.6%)
**All-cause mortality (n, %)**	59 (1.65)	21 (4.0%)	16 (0.6%)	12 (8.1%)	10 (2.0%)

## Discussion

In this study, we investigated the current trends, risk factors, and clinical impact of overall infectious complications occurred in older recipients within the first 6 months following KT using a well-organized nationwide cohort database. Our principal findings are as follows: first, early post-transplant infection was observed more frequently in older kidney recipients than younger recipients; second, the frequency of post-transplant infection was markedly higher within the first month of KT and decreased with time in both older and younger kidney recipients; third, the most common causative pathogen of early post-transplant infection was bacteria and the most frequent site of infection was the urinary tract in both older and younger kidney recipients. Older recipients experienced more mycobacterial infections, co-infections, and multiple site infections compared with younger recipients; fourth, female sex, older donor age, deceased donor, longer duration of hospitalization after KT, and experience of acute rejection before infection were independent factors associated with the development of the early post-transplant infection in older recipients; and finally, early post-transplant infection significantly increases the incidence of rejection, graft loss, and all-cause mortality.

Kidney recipients are at high risk of developing infections due to factors including immunosuppressive status, invasive interventions, and prolonged hospitalization [5, 15]. Despite extensive efforts, post-transplant infection continues to be a major cause of morbidity and mortality after KT and to have significant influence on maintaining allograft function [2, 4]. Although several studies have analyzed the characteristics of post-transplant infection in kidney recipients to provide a basis for the prevention and treatment of infection, few studies have investigated infectious complications after KT in older recipients [12, 13, 16]. In one study been focused on elderly KT patients, 92.4% of the study sample experienced at least one infectious complication during the first year following KT. Infection was a major reason for readmission and increased the risk of future infection in older kidney recipients [16]. The same study group also identified different characteristics of post-transplant infection after KT in older and younger recipients [12].

The incidence of early post-transplant infection in our study population was relatively low compared to that in previous studies [4, 5]. This may be due to differences in the severity of infection defined as the end point. Another previous study that examined hospitalization for infection after KT showed a similar incidence of infection to the present study [17]. In the present study, older recipients experienced more frequent early post-transplant infections and these findings are in line with earlier studies [12, 13, 16]. Older recipients have high risk of post-transplant infection. Previous studies reported that aging could affect changes in the immune system and increase sensitivity to immunosuppresant and infection vulnerability [11, 12]. Older recipients also have a variety of comorbidities that can influence the occurrence of infection and frailty and have negative impacts on health status [12, 18].

In both older and younger recipients, post-transplant infection was markedly increased within the first month after KT and decreased with time. Several studies have reported similar results [19, 20] and it was estimated to be related with the peak use of immunosuppressive drugs in the first month after KT [4, 6]. In our study population, the most common causative pathogens were bacteria, followed by viruses. Our results are similar to those of previous studies, in which bacteria were the most common causative pathogens [4]. However, precise comparisons are difficult because the methods used to distinguish pathogens differ according to the study. As in earlier reports [21, 22], the urinary tract was the most frequent site of early post-transplant infection in our study. Sousa et al. [21] reviewed 1676 kidney recipients and reported that the most common infectious complications were UTIs and the most frequent causative pathogens were *Escherichia coli*, *Enterobacter*, and *Klebsiella*. Other researchers reported that UTIs occurred in 43% of 301 kidney recipients and that the prophylactic use of trimethoprim-sulfamethoxazole (TMP-SMZ) was associated with a reduction in the risk of UTI [22]. In the present study, due to the limitation of the cohort design, we were unable to analyze the causative organisms and the relationship between the use of prophylactic antibiotics and the incidence of early post-transplant infection in detail.

It has been reported that early post-transplant infections are influenced by various factors [4, 19, 23]. Ak et al. [4] demonstrated that female sex, presence of a double J catheter, and duration of hospitalization were independent risk factors of post-transplant infection in kidney recipients. In another study [19], diabetes and type of immunosuppressive regimen were reported to increase the risk of infection after KT. Older recipient age, deceased donor, human leukocyte antigen mismatch, and cold ischemia time were also significant risk factors for post-transplant infection among kidney recipients [23]. Our results were similar: female sex of the recipient, deceased donor, older donor age, longer hospitalization after KT, and experience of acute rejection were indepent risk factors for early post-transplant infection in both younger and older recipients. Given the limitations of our study, the results do not provide a basis for rejecting transplantation in ESRD patients with risk factors for infection. It is necessary to observe more carefully whether infections have occurred and performed routine laboratory tests in kidney recipients with such risk factors.

Post-transplant infection is associated with poor clinical prognosis, such as increased mortality and higher risk of graft loss [2, 24]. We found that early post-transplant infection increased the risks of rejection, graft loss, and all-cause mortality. Therefore, it is necessary to periodically perform clinical tests for kidney function in recipients who have experienced infection to monitor for these clinical events. The underlying mechanisms of associations between post-transplant infection and poor clinical outcomes in kidney recipients are not clearly understood, but these findings are hypothesized to be associated with the enhanced alloreactivity and increased pro-inflammatory response due to infection [25, 26]. Further studies investigating the pathophysiology of infection are needed.

Our study has some limitations. First, the kidney recipients included in this study were predominantly Korean. Considering that post-transplant infection trends may differ according to population origin, socioeconomic circumstances, and post-operative care [24, 27], our findings should be generalized with caution. Second, data regarding species of bacteria or virus were not available in our cohort. Identifying major species of bacteria or viruses that cause post-transplant infection can be of great help in preventing and treating the the infections. We plan to address this limitation in a future study. Third, we did not provide information on prophylaxis for cytomegalovirus or Pneumocystis jiroveci infection due to registry design limitation. Further research is needed on the effects of prophylactic treatment on the incidence of post-transplant infection. Fourth, the younger group is larger than older group, which may associate with the difference in characteristics of the two groups. To reduce this concern, we performed subgroup analysis using 1:1 propensity score-matching. Even after the propensity score matching, the characteristics of post-transplant infection and its impacts on clinical outcomes maintained a similar pattern in each group (data not shown).

## Conclusions

In summary, our results outline current trends and risk factors for the early post-transplant infection after KT in older recipients. The incidence of early post-transplant infection was higher in older kidney recipients than younger recipients, and older recipients experienced more mycobacterial infections, co-infections, and multiple site infections compared with younger recipients. We demonstrated a significant association of early post-transplant infection with clinical outcomes after KT. Careful screening of recipients at high risk for early post-transplant infection and continuous monitoring of recipients experiencing infection are required. Since older recipients have different clinical characteristics from younger recipients, further studies to establish effective treatment strategies for older recipients are needed.

## Supplementary Information

**Additional file 1: Supplementary Table 1.** Cox regression for clinical outcomes according to experience of early post-transplant infection.

## Data Availability

The datasets used and/or analyzed during the current study are available from the corresponding author on reasonable request.

## Abbreviations

ADPKD: Autosomal dominant polycystic kidney disease
ATG: Anti-thymocyte globulin
BMI: Body mass index
CI: Confidence interval
CNS: Central nervous system
DSA: Donor specific antibody
ESRD: End-stage renal disease
GI: Gastrointestinal
HR: Hazard ratio
KOTRY: Korean Organ Transplantation Registry
KT: Kidney transplantation
PCP: Pneumocystis pneumonia
SD: Standard deviation
TMP-SMZ: Trimethoprim-sulfamethoxazole
