# Supplementary material for: Epidemiology, risk factors, and clinical impact of early post-transplant infection in older kidney transplant recipients: the Korean organ transplantation registry study
Source: BMC Geriatr. 2020 Dec 2;20:519. doi: 10.1186/s12877-020-01859-3 (PMC7709316; doi:10.1186/s12877-020-01859-3)
Supplement: Supplementary file 1 — Additional file 1: Supplementary Table 1. Cox regression for clinical outcomes according to experience of early post-transplant infection. [file 12877_2020_1859_MOESM1_ESM.docx]

**Supplementary Table 1. Cox regression for clinical outcomes according to experience of early post-transplant infection**

|  | Recipients | Cardiac event |  | Rejection |  | Graft loss |  | All-cause mortality |  |
| --- | --- | --- | --- | --- | --- | --- | --- | --- | --- |
|  |  | HR (95% CI) | p | HR (95% CI) | p | HR (95% CI) | p | HR (95% CI) | p |
| Crude | younger | 1.140 (0.499–2.603) | 0.756 | 2.063 (1.690–2.518) | < 0.001 | 2.982 (1.678–5.299) | < 0.001 | 6.296 (3.284–12.070) | < 0.001 |
|  | older | 0.595 (0.132–2.685) | 0.499 | 2.291 (1.498–3.505) | < 0.001 | 6.889 (1.723–27.549) | 0.006 | 4.056 (1.752–9.393) | 0.001 |
| Adjusted 1 | younger | 1.140 (0.479–2.612) | 0.757 | 2.124 (1.739–2.594) | < 0.001 | 3.063 (1.720–5.454) | < 0.001 | 6.328 (3.287–12.182) | < 0.001 |
|  | older | 0.580 (0.127-2.540) | 0.481 | 2.285 (1.482–3.525) | < 0.001 | 6.393 (1.577–-25.910) | 0.009 | 4.768 (1.996–11.394) | < 0.001 |
| Adjusted 2 | younger | 1.191 (0.519–2.733) | 0.680 | 2.144 (1.754–2.621) | < 0.001 | 2.998 (1.680–5.350) | < 0.001 | 5.945 (3.078–11.482) | < 0.001 |
|  | older | 0.596 (0.131–2.716) | 0.638 | 2.121 (1.431–3.418) | < 0.001 | 6.599 (1.608–27.085) | 0.009 | 4.931 (2.056–11.825) | < 0.001 |
| Adjusted 3 | younger | 0.861 (0.363–2.044) | 0.734 | 2.016 (1.275–3.188) | 0.003 | 2.360 (1.290–4.317) | 0.005 | 4.697 (2.394–9.212) | < 0.001 |
|  | older | 0.747 (0.153–3.649) | 0.719 | 1.993 (1.622–2.450) | < 0.001 | 7.986 (1.302-28.980) | 0.025 | 2.849 (1.054–7.700) | 0.039 |

Adjusted 1: age, sex, and BMI; Adjusted 2: Adjusted 1 plus hypertension, diabetes mellitus, and cardiovascular disease; Adjusted 3: Adjusted 2 plus re-transplantation, deceased donor, donor age, donor sex, desensitization, ABO incompatibility, presence of DSA, length of hospitalization after KT, induction immunosuppression, calcineurin inhibitors, and steroid.
